# Timeliness of Diagnosing Lung Cancer: Number of Procedures and Time Needed to Establish Diagnosis: Being Right the First Time

**DOI:** 10.1097/MD.0000000000001216

**Published:** 2015-07-24

**Authors:** Akash Verma, Albert Y.H. Lim, Dessmon Y.H. Tai, Soon Keng Goh, Ai Ching Kor, Dokeu Basheer A. A., Akhil Chopra, John Abisheganaden

**Affiliations:** From the Department of Respiratory and Critical Care Medicine, Tan Tock Seng Hospital, Singapore (AV, AYHL, DYHT, SKG, ACK, DBAA, JA); and Johns Hopkins Singapore (AC).

## Abstract

To study number of procedures and time to diagnose lung cancer and factors affecting the timeliness of clinching this diagnosis.

Retrospective cohort study of lung cancer patients who consecutively underwent diagnostic bronchoscopy in 1 year (October 2013 to September 2014).

Out of 101 patients diagnosed with lung cancer from bronchoscopy, average time interval between first abnormal computed tomogram (CT) scan-to-1st procedure, 1st procedure-to-diagnosis, and 1st abnormal CT scan-to-diagnosis was 16 ± 26, 11 ± 19, and 27 ± 33 days, respectively. These intervals were significantly longer in those requiring repeat procedures. Multivariate analysis revealed inconclusive 1st procedure to be the predictor of prolonged (>30 days) CT scan to diagnosis time (*P* = 0.04). Twenty-nine patients (28.7%) required repeat procedures (n = 63). Reasons behind repeating the procedures were inadequate procedure (n = 14), inaccessibility of lesion (n = 9), inappropriate procedure (n = 5), mutation analysis (n = 2), and others (n = 2). Fifty had visible endo-bronchial lesion, 20 had positive bronchus sign, and 83 had enlarged mediastinal/hilar lymph-nodes or central masses adjacent to the airways. Fewer procedures, and shorter procedure to diagnosis time, were observed in those undergoing convex probe endobronchial ultrasound-transbronchial needle aspiration (EBUS-TBNA) (*P* = 0.04).

Most patients exhibit enlarged mediastinal lymph node or mass adjacent to the central airway accessible by convex probe EBUS-TBNA. Hence, combining it with conventional bronchoscopic techniques such as bronchoalveolar lavage, brush, and forceps biopsy increases detection rate, and reduces number of procedures and time to establish diagnosis. This may translate into cost and resource savings, timeliness of diagnosis, greater patient satisfaction, and conceivably better outcomes.

## INTRODUCTION

Lung cancer is the leading cause of cancer-related deaths worldwide.^1^ It has a higher mortality than 4 most common cancers combined, namely breast, prostate, colon, and pancreas.^1^ Unfortunately, two third of the cases are diagnosed in advanced stages.^2–5^ Delays in both presentation and diagnostic workup may contribute to this delay in diagnosis. Cancer researchers have highlighted 2 categories of delay—“access to care” and “hospital processes” related delay—that independently and collectively could impede timely diagnosis.^6–8^ Efforts for early detection by low-dose CT screening have shown mortality reduction,^9^ and smoking cessation has been shown to reduce its incidence.^10^ However, 30–40% of patients with adenocarcinoma being nonsmokers are neither eligible for screening nor can they benefit from smoking cessation.^11–17^ The other factors that affect prognosis in patients with lung cancer are stage, histology, performance status, comorbidity, age, and sex. Most of these factors too are not modifiable.^18^ However, early detection has been shown to be a favorable prognostic factor for survival.^16^ Hence, prompt detection appears to be the only viable option that could potentially have an impact on the outcome of lung cancer patients, and more so on nonsmokers.

Pulmonary medicine service is often the first point of contact in the journey of these patients and comprises the part of the value chain that entails establishing the diagnosis, and clinical stage. Subsequent journey of the patient involves therapy and follow-up, and lies in the department of medical oncology, radiation oncology, thoracic surgery, and palliative medicine and frequently lasts longer than the journey in the department of pulmonary medicine. Since therapy can only begin after the establishment of pathological diagnosis and proper staging, this becomes a rate-limiting step for all downstream processes. Correspondingly, many incidences of dissatisfaction arising from patients with suspected lung cancer originate from delay in diagnosis. Hence, timely detection lends itself as the most ideal candidate for process improvement related initiatives and therefore has been the focus of several investigators in the past.^19–21^

However, limited data exist detailing the number,^22^ sequence of procedures performed, delays and barriers encountered in establishing the diagnosis, relationship between well-tested diagnostic techniques and their impact on timeliness of care for this specific patient group. The wide availability of evidence-based guidelines^23^ provides guidance about the choice of procedures and has improved clinical effectiveness but does not automatically translate into the development of leading-edge care models.^24,25^

In this study, we assessed the number of procedures needed, and delays encountered in the establishment of diagnosis, and factors assisting or impeding timeliness of diagnosis in lung cancer patients who present with nodules or masses on imaging studies.

## METHODS

The current study was part of a quality-of-care initiative performed on patients who consecutively underwent diagnostic bronchoscopy for abnormal CT scan at the endoscopy center of a teaching hospital from October 2013 to September 2014 and eventually received a diagnosis of lung cancer. Retrospective review of demographics, CT findings, type of diagnostic technique employed, pathological result, number of procedures required to reach conclusive diagnosis, and time from 1st CT imaging of the chest to pathological diagnosis, and time from 1st invasive diagnostic procedure to pathological diagnosis. Approval from institutional review board (DSRB) was obtained.

### Case Definitions

We defined the adequacy of the procedure in 2 ways.

Inadequate procedure: When visible endobronchial lesion amenable to mucosal biopsy was present but biopsy was not done; when positive bronchus sign was present but trans bronchial biopsy was not done; or when enlarged mediastinal/hilar lymph nodes or masses adjacent to the central airways amenable to convex probe endobronchial ultrasound-transbronchial needle aspiration (EBUS-TBNA) were present but EBUS-TBNA or conventional TBNA was not done.

### Inappropriate Procedure

When a site other than the one that would have provided the highest disease stage along with the diagnosis was used to establish the diagnosis (eg, patient with a suspicious malignant pleural effusion but thoracentesis or pleural biopsy not undertaken as 1st procedure).

### Data Analysis

We used software (SPSS, version 17; SPSS Inc, Chicago, IL) for all statistical analyses. The results were compared using a Wilcoxon two-sample test or Fisher exact test. *P* values were two sided and considered indicative of a significant difference if less than 0.05.

## RESULTS

One hundred one patients out of those who underwent diagnostic bronchoscopy were diagnosed with lung cancer in the study period. Adenocarcinoma was the most common subtype seen in 44 (43.5%) patients and more than half (54.5%) of these were never smokers. When compared with nonadenocarcinoma group, more patients were nonsmoking females with concomitant absence of scarring (*P* = 0.04) or emphysema (*P* = 0.04) on CT scan in adenocarcinoma group (Table 1).

**TABLE 1:**
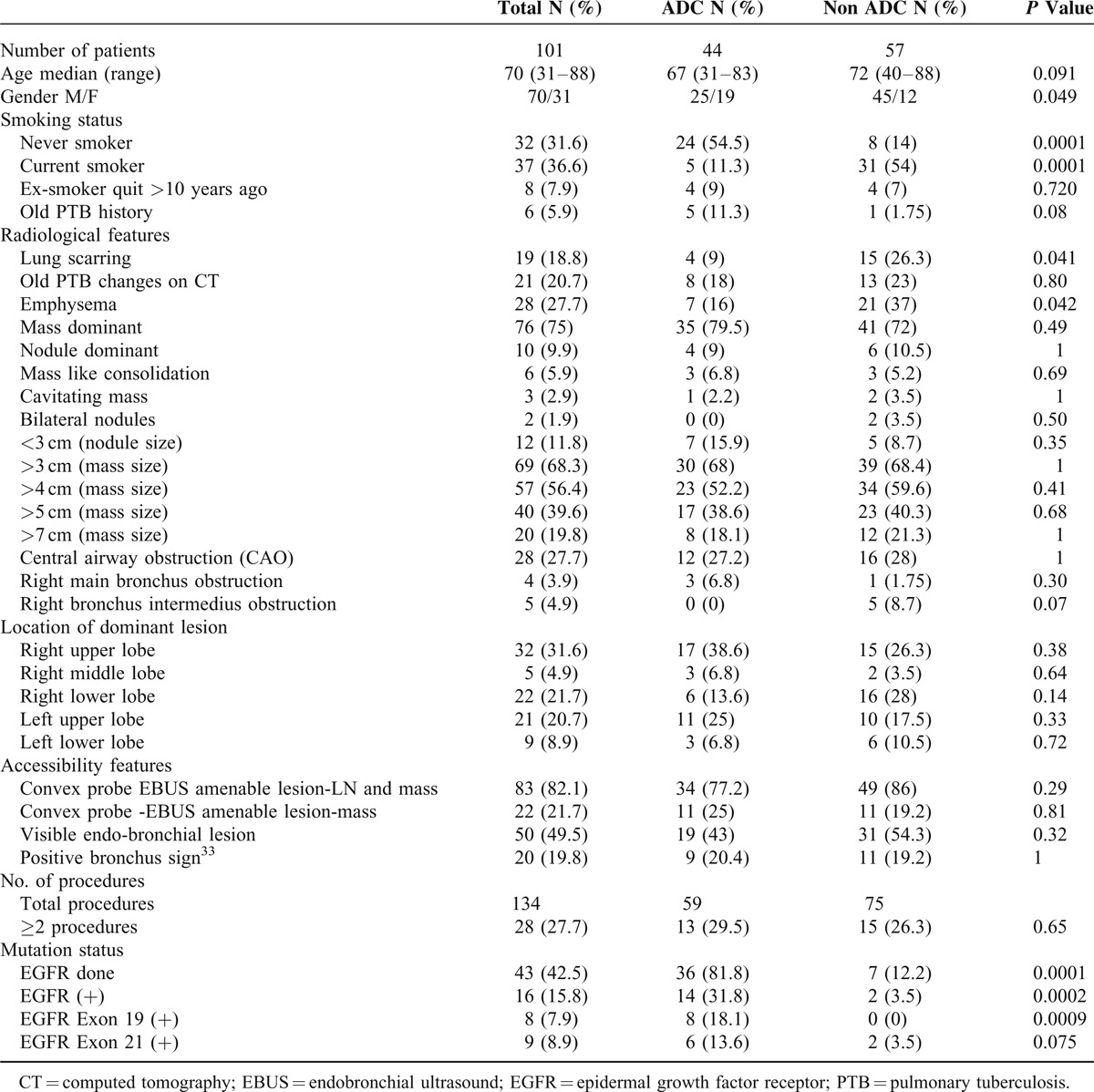
General Characteristics of the Patients and Subgroup Analysis of Adenocarcinoma (ADC) and Non-Adenocarcinoma (Non ADC) Patients

### Number and Type of Procedures

One hundred thirty four procedures were done in 101 patients to establish the diagnosis. In 72 patients, diagnosis could be established by single procedure, whereas 29 patients (28.7%) required multiple procedures (n = 63). The types of procedures performed were bronchoalveolar lavage (BAL, 103), bronchial biopsies (85), EBUS-TBNA (23), transthoracic needle aspiration (TTNA, 12), wedge resections (5), mediastinoscopies (2), and thoracentesis (3). No procedure-related complication was noted except in case of TTNA, 6 (50%) of which were complicated by pneumothorax.

### Time to Establish Diagnosis

**FIGURE 1:**
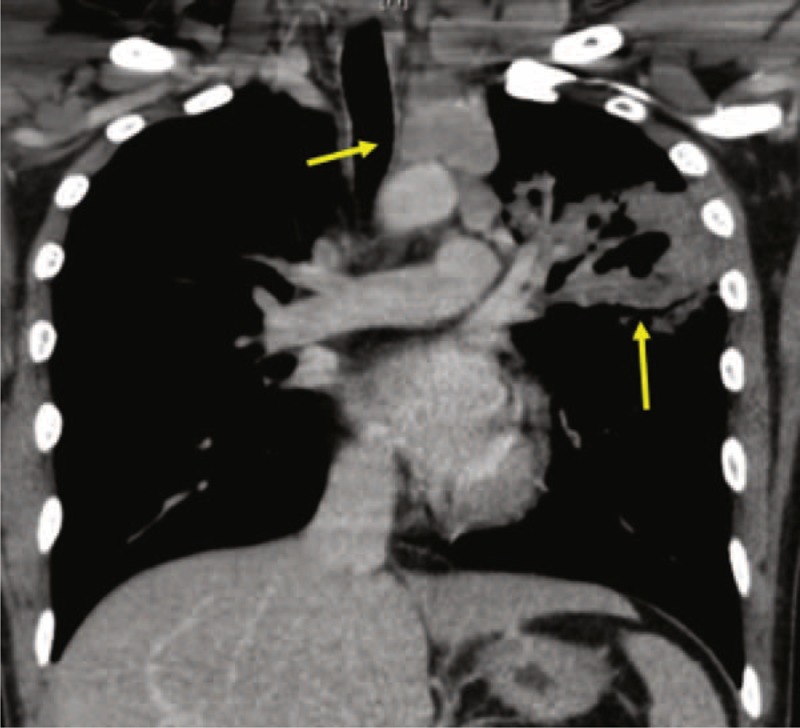
A representative case of patients with concomitant peripheral and central lesions undergoing both bronchial washing with biopsy, and EBUS-TBNA. Higher diagnostic yield was seen with EBUS-TBNA. EBUS-TBNA = endobronchial ultrasound-transbronchial needle aspiration.

Average time interval between 1st abnormal CT scan to 1st procedure, 1st procedure to diagnosis, and CT scan to diagnosis was 16 ± 26, 11 ± 19, and 27 ± 33 days, respectively. All these intervals were significantly longer in the group requiring repeat procedures (*P* = 0.04, *P* = 0.001, and *P* = 0.001 respectively). Requiring 2 or more procedures was identified as the predictor of prolonged (>30 days) CT scan to diagnosis time in multivariate analysis (Figure 1).

### Factors Assisting Timeliness

**FIGURE 2:**
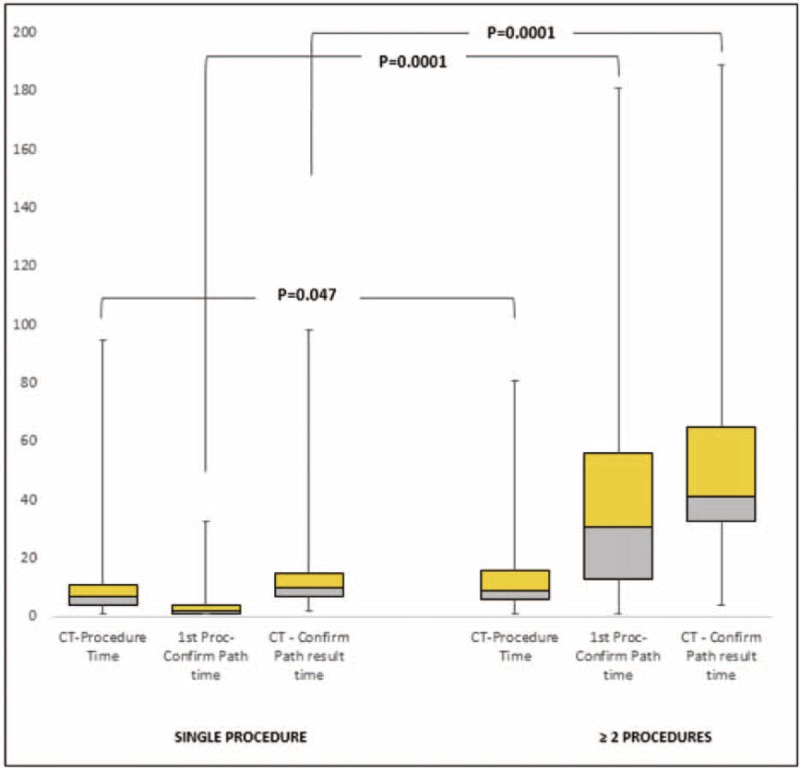
Patients with ≥ 2 procedures had prolonged CT scan-to-confirmation of diagnosis time. CT = computed tomography.

Comparison of diagnostic yield among bronchoscopic procedures revealed the yield of EBUS-TBNA to be significantly higher than BAL or washings (*P* = 0.001). EBUS-TBNA cytology was more diagnostic (48%) than cytology from BAL or washings (23%, *P* = 0.03) (Table 2). In the group of patients who had concomitant peripheral and central lesions (Figure 2), and underwent both bronchial washing with biopsy, and EBUS-TBNA, TBNA showed a trend for higher diagnostic yield 37% versus 74% (*P* = 0.02), respectively (Table 3). Fewer procedures were required to establish the pathological diagnosis in the EBUS-TBNA group versus bronchoscopy group (*P* = 0.006). A third of the patients required second or more procedure in the bronchoscopy group as compared to only 1 patient requiring it in the EBUS-TBNA group. In those who underwent EBUS-TBNA, the procedure to pathological diagnosis time was shorter as compared to bronchoscopy group. Fewer patients in EBUS-TBNA group had more than a week (4.3%) or 2 weeks (4.3%) interval between procedure and establishment of diagnosis as compared to 29.4% and 24% of patients in the bronchoscopy group (*P* = 0.01, *P* = 0.03), respectively (Table 4).

**TABLE 2:**
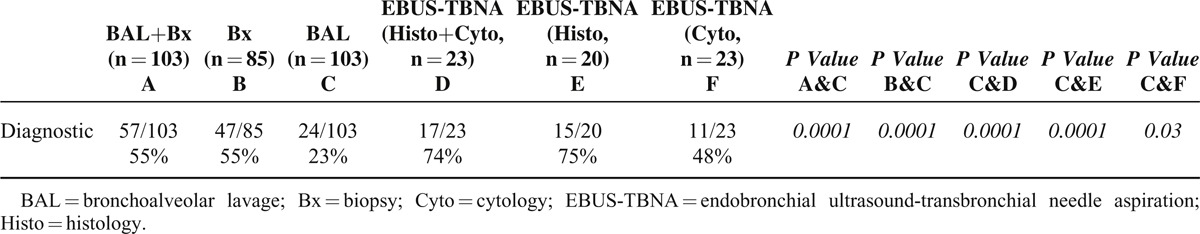
The Yield of Various Bronchoscopic Procedures in the Diagnosis If Lung Cancer

**TABLE 3:**
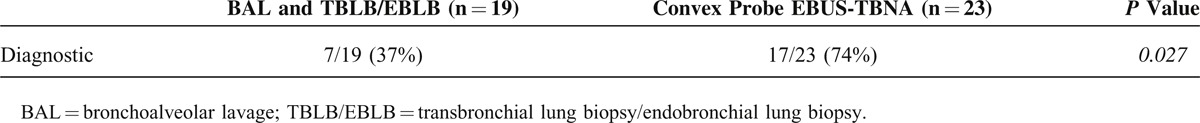
The Yield of Combined BAL/TBLB with Convex Probe EBUS-TBNA in the Diagnosis of Both Central and Peripheral Malignant Lesions

**TABLE 4:**
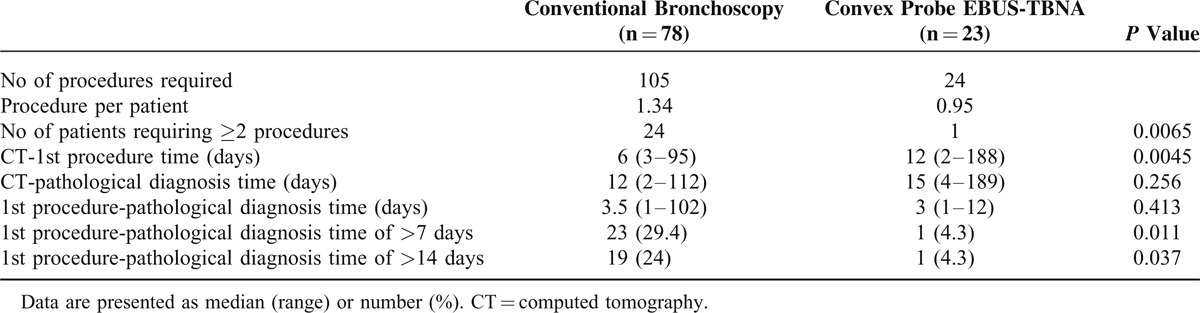
Number of Procedures and Time Interval Between Procedure and Histological Diagnosis of Lung Cancer

### Factors Impeding Timeliness

Searching for the reasons behind the need for repeat procedures showed inadequacy of 1st procedure to be the most common cause seen in 13.8% of patients. The second most common cause in patients requiring ≥2 procedures was inaccessibility of the lesion seen in 9% of patients. These patients did not have visible endo-bronchial lesion, airway leading to the lesion, or mediastinal/hilar lymph node or mass amenable to convex probe EBUS-TBNA (Table 5).

**TABLE 5:**
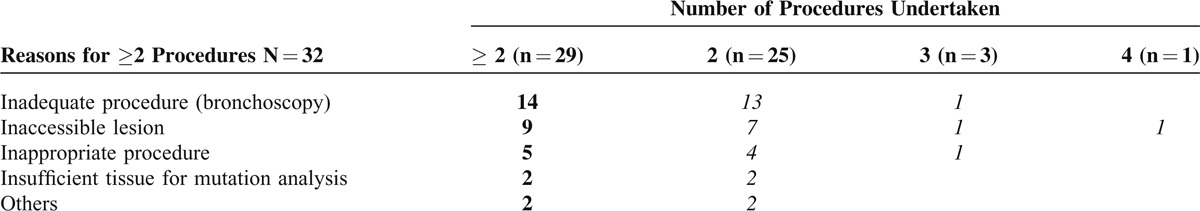
Reasons for Requiring 2 or More Procedures (n = 29) Before Making a Histological Diagnosis of Lung Cancer

## DISCUSSION AND CONCLUSION

Our main finding was that most patients exhibited enlarged para-tracheal and peri-bronchial lymph node or masses adjacent to the central airways that were accessible by convex probe EBUS-TBNA. Combining it with the conventional bronchoscopic techniques such as BAL, brush and forceps biopsy, increased detection rate and reduced number of procedures and time needed to establish the diagnosis of lung cancer.

Most of the factors known to affect prognosis of lung cancer are not modifiable. Although low-dose CT screening and smoking cessation has shown benefit, these measures are not applicable to all lung cancer patients such as nonsmokers.^9,10^ Nonsmokers constituted 31.6% of the nonsmall cell lung cancer (NSCLC) patients in our population, and among those with adenocarcinoma, this proportion was 54.5%, consistent with previous studies.^12,15,16^ This exemplifies that timely diagnosis is the only most widely applicable modifiable factor for adding value to their care.

The duration between 1st procedure to diagnosis and 1st abnormal CT imaging to diagnosis was inappropriately long in 28% and 40% of patients, respectively. The time taken to establish the diagnosis from the point of 1st invasive procedure and from the 1st contact with pulmonary unit have been identified as quality indicators. Benchmarks have been established based on the expert consensus and evidence-based guideline recommending not to exceed this time beyond 7 and 20 days, respectively.^24^ In the present study, average time interval between 1st invasive procedure to diagnosis and 1st CT scan to diagnosis was 11 ± 19 and 27 ± 33 days, respectively. These long intervals correlated with the number of procedures undertaken to establish the diagnosis. Those with multiple procedures had longer intervals. The positive correlation between repeat procedures and prolonged intervals between CT scan or 1st invasive procedure and diagnosis indicated that the delay in diagnosis can be eliminated by minimizing the number of procedures.

The most common cause of requiring repeat procedures was inadequate 1st procedure. Half of the patients who required 2 or more procedures either did not undergo bronchial biopsy or convex probe EBUS-TBNA despite bronchus sign,^35^ or mediastinal lymph node or central mass amenable to convex probe EBUS-TBNA, respectively. Those patients who underwent EBUS-TBNA as the 1st procedure, number of procedures and time needed to establish diagnosis was shorter. Those patients who underwent EBUS-TBNA as 2nd or 3rd procedure in the group requiring repeat procedure, did not require any procedure thereafter. It is noteworthy that 82% of the patients had lesion accessible by convex probe EBUS in our cohort either in the form of mediastinal/hilar lymph nodes, or intraparenchymal lesions both of which could be aspirated by convex probe EBUS-TBNA,^25–29^ but it was only done in 22% (n = 23) of the patients. It is conceivable that had these patients undergone EBUS-TBNA, the number of procedure required may have been less, and the delay in the diagnosis may have been reduced in a larger number of patients.

The high yield of convex probe EBUS-TBNA and its translation into reduced number of procedures and time to diagnosis is understandable. It has been established that among the bronchoscopic techniques for lung masses, needle techniques provide a higher diagnostic yield than BAL, brush, or forceps biopsy.^30–32^ The potential for *needle* to bypass surface and sample viable tumor or lymph-nodes beneath the trachea and bronchi is the possible explanation.^30–32^ In extrinsic compression, conventional procedures using brushing or biopsy tend to sample mainly the surface rather than deep within the lesion. Convex probe EBUS-TBNA seemed to be superior in our patients for 3 reasons. First, most patients (82%) had a lesion accessible via convex probe EBUS-TBNA. Second, it provided nodal staging. Third, no complications were noted. On the other hand, 50% of the patients (higher than published literature)^33,34^ who underwent TTNA developed pneumothorax in our cohort. High rate of pneumothorax, although not life threatening most of the times, still adds economic burden by necessitating hospitalization for observation or chest tube insertion and defeats the measures put up in place to reduce length of stay in the hospitals.

The second most common cause in patients requiring repeat procedures was inaccessibility of the lesion. In a third of patients requiring repeat procedures, no lesion accessible by bronchoscopy or EBUS-TBNA was found. These patients were genuinely challenging and assisted technologies such as navigation bronchoscopy may have helped.

Our study has limitations of a retrospective single-center study susceptible to information and institutional clinical practice bias limiting its generalizability. However, the strength of the study is that it emphasizes on timeliness*—*an ignored, but as important as diagnostic and therapeutic aspect of lung cancer management.

In conclusion, several clinical and organizational factors have been associated with delayed diagnosis of lung cancer.^36–38^ Our study confirmed that failure of first diagnostic procedure to yield the diagnosis correlates with the diagnostic delay. This necessitates “rework” at the expense of cost, time, resources, and exposure of the patient to risk. It also lowers patient satisfaction and additionally, some patients become too sick, or give up and decline subsequent procedures. All these issues can be minimized by emphasizing on the appropriateness of the 1st procedure. Since most patients with lung cancer have concomitant para-tracheal or peri-bronchial convex probe EBUS-TBNA amenable lesion in addition to primary mass lesion in the parenchyma, recognition of these lesions and greater utilization of convex probe EBUS-TBNA by combining it with BAL and bronchial biopsy (transbronchial or endobronchial) can provide the conclusive diagnosis more frequently than bronchoscopic biopsy alone. Basing decisions regarding diagnostic procedure on the theme of *“*first most suitable procedure in order to be right the first time,” and greater adoption and integration of convex probe EBUS-TBNA in the diagnostic work up may help to improve timeliness of care in lung cancer. This has a potential to translate into cost, time, resources, and risk sparing benefits, along with greater patient satisfaction, and conceivably better outcomes.
